# A Practical Treatise on Materia Medica and Therapeutics

**Published:** 1893-08

**Authors:** 


### BOOK NOTICES.

A Practical Treatise on Materia Medica and Therapeutics, with Especial Reference to the Clinical Application of Drugs. By John V. Shoemaker, A. M., M. D. Second edition. Revised. In two royal octavo volumes. Volume I, 353 pages: Devoted to Pharmacy, General Pharmacology, and Therapeutics and Remedial Agents not Properly Classed with Drugs. Volume II, 680 pages: An Independent Volume upon Drugs. Volume I, in cloth, $2.50 net; sheep, $3.25 net. Volume II, in cloth, $3.50 net; sheep, $4.50, net. Philadelphia: The F. A. Davis Co., Publishers, 1914 and 1916 Cherry Street.

This is the finest and latest treatise upon the materia medica of the old school of medicine. The first volume gives not alone the pharmacy of drugs, but also the methods of applying electricity in paralysis, gynæcology, dermatology, the removal of superfluous hair, its use in throat and nose diseases, and electrical illumination of internal organs. Massage and the rest cure receive notice, as well as ozone and hydrogen peroxide. It would be quite impossible to give a catalogue of all the remedial measures treated of in this volume, but whoever is interested in the subject will have his desire for information satisfied by providing himself with a copy of the book.

The second volume contains a complete encyclopedia of all drugs used in therapeutics, arranged in alphabetical order.

An index to drugs and preparations, and a clinical index complete the volume. The two volumes together constitute a reliable and satisfactory book of reference.

